# Did You Just Cough? Visualization of Vapor Diffusion in an Office Using Computational Fluid Dynamics Analysis

**DOI:** 10.3390/ijerph19169928

**Published:** 2022-08-11

**Authors:** Mohammad Al-Rawi, Ahmed M. Al-Jumaily, Annette Lazonby

**Affiliations:** 1 Centre for Engineering and Industrial Design (CEID), Waikato Institute of Technology, Hamilton 3240, New Zealand; 2 Institute of Biomedical Technologies (IBTec), Auckland University of Technology (AUT), Auckland 1010, New Zealand; 3 Faculty of Business and Economics, The University of Auckland, Auckland 1010, New Zealand

**Keywords:** infectious respiratory diseases, ultraviolet germicidal irradiation (UVGI), dehumidifier, CFD modelling, Discrete Phase Model (DPM)

## Abstract

Awareness of indoor air quality (IAQ) in crowded places such as schools and offices has increased since 2020 due to the COVID-19 pandemic. In addition, countries’ shifting away from containment and towards living with COVID-19 is expected to increase demand for risk mitigation via air-purification devices. In this work, we use Computational Fluid Dynamics (CFD) analysis to investigate the impact of adding an air-purification technology on airflow in an enclosed space. We model a Polyester Filter and UV light (PFUV) dehumidifier in an office with two occupants: one infected with an airborne infectious disease, such as COVID-19; and the other uninfected. We compare three cases where the infected occupant coughs: with no device, and with the device at two different orientations. We construct a CFD model using ANSYS^®^ 2021 Fluent and the Discrete Phase Model (DPM) for the particle treatment. Thermal comfort is assessed using the Testo 400 IAQ and comfort kit. We find that both the device operation and the placement alter the airflow contours, significantly reducing the potential for the uninfected occupant to inhale the vapour expelled by the infected occupant, potentially impacting the likelihood of disease transmission. The device improved thermal comfort measured by Predicted Mean Vote (PMV), Predicted Percentage Dissatisfied (PPD).

## 1. Introduction

The COVID-19 pandemic which was declared in 2020 has dramatically increased attention on the indoor air quality (IAQ) in spaces where individuals are required to “share air”, that is, enclosed spaces in which individuals must inhale air that has been circulated around a closed system containing exhaled air from other individuals sharing that space. At the beginning of the pandemic, schools, government institutions and private businesses responded primarily by shifting to remote delivery/working, where possible. Now those same organizations are largely returning to either partially or fully in-person attendance/work and must face the issue of whether these places in which students, workers or members of the public are required to gather have satisfactory IAQ. This is all the more pressing as the pandemic has dramatically increased individuals’ awareness of risks of airborne virus transmission (particularly of SARS-CoV-2) [1,2,3].

Respiratory diseases, such as influenza and COVID-19, are transmitted between individuals via aerosols: droplets of much larger diameter than the virion itself. These emit from the infected person via a cough or expiration (exhaling) to potentially be inspired (inhaled) by other occupants in close proximity, allowing for virus transmission. Aside from pathogen transfer, other factors such as particulate matter load, are also a concern for air quality in general (and IAQ in particular), according to the World Health Organization (WHO), which is issuing an update to its guidelines on air quality [4]. Furthermore, there are other factors that matter to occupants of an indoor space, such as thermal comfort parameters, which also should be taken into account by researchers and in the formation of standards [5].

Airborne transmission of an infectious disease has been numerically investigated based on the outbreak of SARS in 2003 [6]. Many mathematical and computational models have been conducted using a virtual thermal mannequin [7,8,9,10,11,12]. This method allows for adjustment of the shape of the geometry to investigate the airborne spread of expiratory droplets [9]. This airborne spread of droplets (vapor) carrying the virus increases the risk of infection to those currently uninfected occupants who are also breathing that air. Therefore, the last 20 years have seen significant exploration of efficient ventilation technologies to dually achieve a comfortable indoor climate for occupants as well as reducing the risk of cross-infection and contaminant spread in closed indoor environments [13].

An experimental investigation into the dispersion of contaminants conducted by Olmedo et al. simulated two thermal mannequins in a room with a ventilation device source at the ceiling. The flow direction could be altered to be downward or upward. The “infected” mannequin was set at 0.75 L/exhalation, whilst a gas tracer (N_2_O) detected airborne cross-infection. Under these settings, the greatest exposure to cross-infection occurred when the distance between the mannequins was less than 0.5 m [13].

The trajectories of particles in the mouth of a room occupant were investigated computationally using ANSYS-Fluent 13. The study altered the air velocity to identify how the size of the particles inhaled differed depending on the velocity of inhalation. The traced particles’ trajectories are presented under low air velocity (0.1–0.4 m/s), at rest inhalation (7.5 m/s), moderately paced breathing (20.8 m/s) and heavy breathing (50.31 m/s). Only under the lattermost velocity (heavy breathing) were the large particles having 100–116 μm diameter inhaled into the mouth [14].

Thermal comfort parameters lie under the umbrella of Indoor Environmental Quality (IEQ) and have been investigated in the literature using Computational Fluid Dynamics (CFD) methods along with experimental field data [14,15,16,17]. The parameters that are of interest include: the predicted mean vote (PMV); the predicted percentage dissatisfied (PPD), along with indoor temperature and indoor relative humidity and fall under ASHRAE Standard 55–2017.

CFD evaluations of a combined HVAC and smart window-integrated ventilation system have been used to investigate the system’s performance in terms of thermal comfort and IAQ [15]. The model employed Reynolds-averaged Navier–Stokes (RANS) equations with a k−ε turbulence flow regime solved in STAR-CCM+. The computational model performed well, with an average error of 5% for velocity and temperature compared to field data measurements. Li and Tong also recommended the use of the RANS and a k−ε  turbulence flow regime for the study of thermal environments in real time. [16]. Shan et al. included occupants’ characteristics, such as dimensions and calorific value (75 W/person) in the boundary conditions for the CFD analysis, along with the boundary conditions for the room (an overall thermal transmission coefficient (U) with 0.2 W/m^2^k for the external wall and 3 W/m^2^k for the window) [17]. The simulation model was validated using a thermo-flow sensor with respect to the indoor temperature and velocity. Gao et al. used CFD analysis to assess the degree to which pollutant exposure was reduced and thermal comfort adjusted based on a personalized ventilation design [18]. They used a gas tracer to assess IAQ parameters, examining the emission of volatile organic compounds (VOCs) via diffusion from a painted ceiling and sidewall, as well as the dust discharged from the floor. They found that the air movement was especially important at the head level, to achieve an acceptable level of thermal comfort. Licina et al. experimentally investigated the effect of body posture and boundary layer temperature, and method of breathing, on air the temperature, using a mannequin. Their results confirmed the assumption that exhaling via the mouth will reduce air temperature in front of the chest, whereas breathing via the nose did not affect the temperature, due to the physical properties of the jet’s flow from the nose [19]. Additionally, the CFD models presented in the literature show an average error between 3.61% and 9%, with variations according to the software packages used or the mesh quality; moreover, this kind of application requires significant processing power.

Recent CFD studies investigating the distribution of droplets containing SARS-CoV-2 focused on the effect of an infected patient’s coughing in various shared (multi-occupant) settings such as classrooms [20], health care facilities [21] and urban seating configurations [22]. Transportation of the virion through ventilation systems has also been examined, using CFD methods [23]. These studies therefore provide excellent data for CFD modelling related to inhalation and exhalation of an infected occupant (the contaminant source) and healthy occupants in both closed [20,21] and open [22] systems.

To the best of our knowledge, CFD simulation of the effect of using an air-purification device which also has the effect of altering the air-flow contours has not been investigated. Further, recent studies which focused on the concern to public health posed by the COVID-19 pandemic [24,25] do not approach this from the CFD perspective. In this work, we use CFD to examine the air-flow behavior in a room shared by two occupants, in which an air decontamination device, called a Polyester Filter and UV light (PFUV) dehumidifier is used. This PFUV dehumidifier combines HEPA filtration with Ultraviolet Germicidal Irradiation (UVGI) lights and dehumidification to affect the IEQ in a room. The paper assesses the impact of operating the PFUV dehumidifier device in an office shared by two occupants: one infected with a transmitted disease (such as COVID-19) and the other uninfected. We investigate the effect of altering the location of the device and the location of the occupants to identify the behavior of the air flow, and the passage of air from the infected to the uninfected occupant, under these different scenarios. The CFD model is validated with experimental data to generate a model within ANSYS to predict any future design. The degree to which the PFUV device cleanses the air of infection is not examined in this article as this is reserved for future work.

## 2. Materials and Methods

### 2.1. Geometry Model

The field data were collected in a residential house located in a rural part of North Waikato, New Zealand. Air quality in the region is considered above average, (low concentrations of particulate matter (PM_2.5_) compared to urban/suburban environments) [3]. The room under investigation is a “home office”. In this office we added a PFUV (DB48WH-NZGC dehumidifier, General Electric Company Limited (GEC Ltd.), Boston, MA, USA) to address IAQ elements such as reducing the relative humidity and purifying the air (using two 8 W germicidal fluorescent T5 UV lights) and a particulate matter filter (using CityPleat-200, manufactured by Camfil AB Stockholm, Sweden and supplied by Camfil NZ Limited, Auckland, New Zealand). The filter is based on ASHRAE MERV A 7A combining synthetic fiber and broad-spectrum carbon. The air passes through the UV light rays to denature microbes. The filters work to trap particulate matter from the air. This device was tested in the office and reduced the dampness readings whilst increasing the room temperature during low outdoor temperatures (below 7 °C) [3]. The technical specifications, including energy consumption, are available in [3].

**Figure 1:**
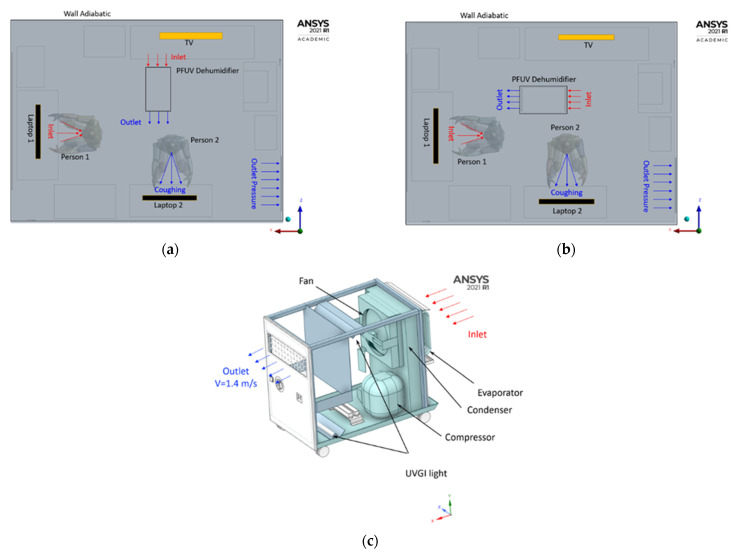
The location of the PFUV dehumidifier: (**a**) top view scenario A; (**b**) top view scenario B; and (**c**) the PFUV dehumidifier (iso-view).

Two scenarios for placing the PFUV dehumidifier in an office shared by two occupants are considered: Figure 1a with the outlet faces Person 2, while in Figure 1b, the outlet faces Person 1. We investigate how this differentiated placement of the PFUV device affects the air-flow contours in the room. Person 1 is uninfected, whilst Person 2 is infected; therefore Person 1 is inhaling through the nose, while Person 2 is exhaling with force (i.e., coughing) through the mouth.

### 2.2. Computational Model and Boundary Conditions

**Figure 2:**
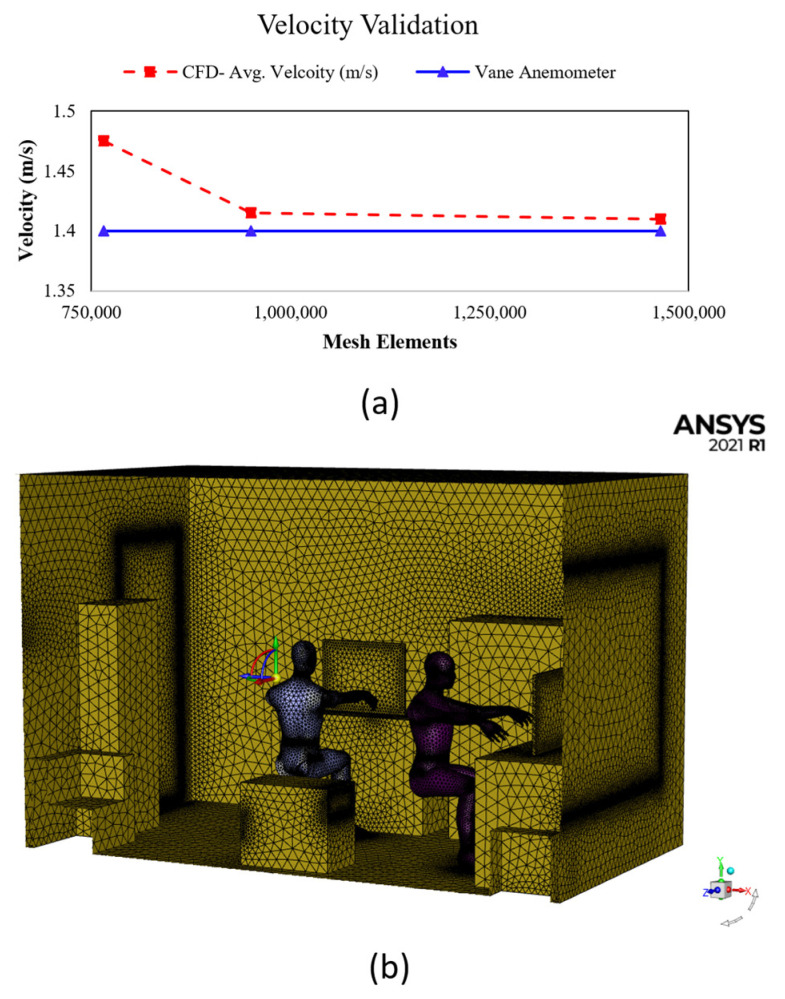
(**a**) Velocity validation, the CFD average velocity (m/s) and the Vane Anemometer measurements; and (**b**) the iso-view mesh for the office showing Person 1 and Person 2 and the PFUV in scenario B.

The 3D model is constructed using Design Modeler in ANSYS, as shown in Figure 2a, by implementing field data measurements. The geometry is prepared and then a suitable mesh is chosen within the ANSYS workbench. In our model we assume the office is an adiabatic system and model the indoor conditions with the assumption that the fluid temperature is 14 °C and relative humidity is 65%, which is not isothermal. The model for this process consists of solving the following governing equations:

The three-dimensional continuity equation for incompressible flow:

(1) $$\frac{\partial \rho }{\partial t}+\frac{\partial \left(\rho u_{i}\right)}{\partial x_{i}}=0$$

where ρ is the density of air, a constant, and $u\;\mathrm{is}\;\mathrm{the}\;\mathrm{velocity}\;\mathrm{vector}\;\mathrm{in}\;\mathrm{three}\;\mathrm{dimensions},\;u_{x},u_{y}\;\mathrm{and}\;u_{z}$.

The three-dimensional momentum equation:

(2) $$\frac{\partial \left(\rho u_{i}\right)}{\partial t}+\frac{\partial \left(\rho u_{i}\right)}{\partial x_{i}}+\frac{\partial \left(\rho u_{j}u_{i}\right)}{\partial x_{i}}=-\frac{\partial p}{\partial x_{i}}+\frac{\partial \tau_{ij}}{\partial x_{i}}-\rho g_{j}+F$$

where p is the pressure, τ is the viscous stress tensor, $\rho g_{i}$ represents the force exerted by gravity in the negative y-direction and F is the body force.

The three-dimensional energy equation:

(3) $$\rho \frac{DE}{Dt}=\left[-\nabla \left(pu\right)\right]+\left[\frac{\partial \left(u_{x}\tau_{xx}\right)}{\partial x}+\frac{\partial \left(u_{x}\tau_{yx}\right)}{\partial y}+\frac{\partial \left(u_{x}\tau_{zx}\right)}{\partial z}+\frac{\partial \left(u_{y}\tau_{xy}\right)}{\partial x}+\frac{\partial \left(u_{y}\tau_{yy}\right)}{\partial y}+\frac{\partial \left(u_{y}\tau_{zy}\right)}{\partial z}+\frac{\partial \left(u_{z}\tau_{xz}\right)}{\partial x}+\frac{\partial \left(u_{z}\tau_{yz}\right)}{\partial y}+\frac{\partial \left(u_{z}\tau_{zz}\right)}{\partial z}\right]+\nabla \cdot \left(k\nabla T\right)+S$$

where *E* is energy, *T* is Temperature, and *S* is the heat source. These equations are solved using the 3D general transport equation, which in our model represents the transient convection diffusion source [5].

(4) $$\frac{\partial \left(\rho \emptyset \right)}{\partial t}+\frac{\partial \left(\rho u_{j}\emptyset \right)}{\partial x_{j}}=\frac{\partial }{\partial x_{j}}\left(\Gamma \frac{\partial \emptyset }{\partial x_{j}}\right)+S_{\emptyset }$$

where the dependent variable ∅ represents temperature and velocity ($u_{x},\;u_{y},\;u_{z}$) in transient time (t), Γ is the diffusion coefficient, the partial derivative $\frac{\partial \left(\rho \emptyset \right)}{\partial t}$ reflects the intensive variable change ∅ (velocity and temperature) with respect to time in a control volume, $\frac{\partial \left(\rho u_{j}\emptyset \right)}{\partial x_{j}}$ is the convective term to determine the flow rate of variable ∅ caused by the velocity $u_{j}$, $\frac{\partial }{\partial x_{j}}\left(\Gamma \frac{\partial \emptyset }{\partial x_{j}}\right)$ is the diffusion term for the variable ∅ within the control volume of the office and $S_{\emptyset }$ is the general source term.

The Discrete Phase Model (DPM) was employed. This models a water (H_2_O) droplet ejected from the mouth of the unhealthy person. Under ANSYS Fluent, we activated the energy equation. Under the viscous model, we used the k-epsilon (2 eqn) and activated the realizable, which is the best method for studying separated flow with complex second-flow features. Under the near wall treatment, we activated the standard wall functions to study the flow behavior near the wall. We activated the species transport model and used the diffusion energy source. To create the mixture of air and H_2_O we used incompressible-ideal gas density. Then, we set the Discrete Phase Model using interaction with a continuous phase to create the injection with a material water (liquid) and evaporating species as H_2_O, using injection type to the surface on the mouth of the coughing person. For the particle treatment, we set this to unsteady particle tracking and the boundary condition to trap type. The injection model is set as inert as water (liquid) from the mouth of the coughing person with the following values for velocity (0–22.352 m/s and temperature 34 ℃).

The boundary conditions are based on the physical parameters of the office (dimensions) as shown in Table 1 and the flow data, which is measured using a Vane Anemometer (Testo 410–2, a plastic impeller sensor operating with 0.1 m/s resolution and an accuracy of ± 0.2 m/s + 2% of the reading with range 0.4 to 20 m/s) as shown in Table 2. The velocity (m/s) arises from the operation of the PFUV dehumidifier. Additionally, the Testo 410-2 sensor provides readings for temperature (measuring range −10 to +50 ℃, accuracy ± 0.5 ℃) and relative humidity (measuring range 0–100%, accuracy ± 2.5% (5–95%RH) and 0.1%RH resolution). The field data collected for the room in which the electrical devices operated (laptops, screen, lights, etc.) are provided in Table 1. The data for Person 1 and Person 2 are obtained from the literature [5,14].

### 2.3. Grid Indpendence and Validation

The generated office dimensions are 3900 × 2500 × 2900 mm, as shown in Table 1. Then we progressively adjusted the element size (0.03–0.01 m) to achieve an acceptable mesh. We also added the inflation layer to focus on the model for detailed CFD analysis. In this study, the inflation boundary layer was set to total thickness, with number of layers equal to three, growth rate equal to 1.1, a maximum thickness of 0.05 m and inflation algorithm set to “Pre”. Table 3 shows the element sizes. The mesh elements for the PFUV’s outlet velocity were validated using the Vane Anemometer (Testo 410-2).

Mesh independence tests were repeated until the velocity of 1.4 m/s (with an average error 2.3%) was reached; this is in the acceptable range, as depicted in Figure 2a. The office dimensions are 3900 × 2500 × 2900 mm with office furniture, equipment and two occupants generated under ANSYS Design Modeler. These parts are converted to one part using the Boolean option to prepare them for simulation under ANSYS Fluent. Setting up the inlet and outlet boundary conditions will help us to use the watertight geometry type of the workflow. This assists in adding local sizing for mesh surfaces of interest, such as the mouth, nose and PFUV inlet and outlet using the face size control. Then, the geometry under ANSYS Fluent Meshing is described, consisting of both fluid and solid regions. The final step is to create the volume mesh using the smooth-transition offset method filled with a tetrahedral mesh type. This resulted in a mesh element size of 1,465,125, which is indicated as acceptable by the literature [7,15,17], where sensitive areas, such as inlet and outlet regions and edges have a finer mesh, as shown in Figure 2b, to obtain accurate results. The duration of the calculation process was 30 h (computer specifications: Intel^®^ Core^TM^ i7-8750H CPU @ 2.20GHz 2.21 GHz and 31.9 GB usable, 64-bit Operating System).

## 3. Results and Discussion

### 3.1. Velocity Contours

**Figure 3:**
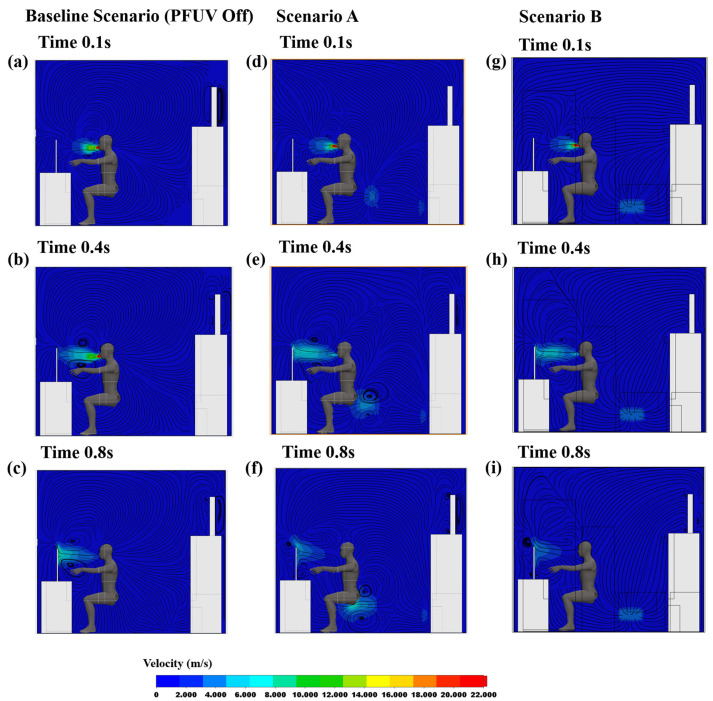
Velocity contours showing Person 2 coughing: Baseline Scenario (PFUV Off) (**a**) 0.1 s, (**b**) 0.4 s, (**c**) 0.8 s; Scenario A (**d**) 0.1 s, (**e**) 0.4 s, (**f**) 0.8 s; and Scenario B (**g**) 0.1 s, (**h**) 0.4 s, (**i**) 0.8 s.

In order to trace the air-flow behavior between the infected and uninfected room occupants (Person 2 and Person 1, respectively), we generated the velocity contour profiles for both scenarios (different locations of the devices) using transient analysis. Figure 3 shows the velocity contours on a mid-sagittal plane section (representing a longitudinal division of the body into left and right sides). Three scenarios are shown: with the device switched off (the Baseline Scenario), with the outlet of the operating device facing Person 2 (Scenario A) and with the outlet of the operating device facing Person 1 (Scenario B). For each scenario, the contour map is provided at three different time phases of the coughing action: 0.1 s, 0.4 s and 0.8 s.

Figure 3 shows the spread of the coughing on the mid-sagittal plane. The red/orange blob represents the air-stream emitted with force from the infected person’s mouth (Person 2), who is coughing on the monitor, and this dissipates later in the cough (at 0.8 s) into a lower force (shown in light blue). Figure 3a–c show the Baseline Scenario, where the cough generates significant airflow in and of itself, but there is no interaction of the PFUV device (it is switched off in that scenario). In Scenario A (placing the PFUV outlet facing the back of Person 2) the velocity contours of Person 2 coughing (vapor) can be seen, while the PFUV alters the air-flow contours when delivering fresh air to the back of Person 2, as shown in Figure 3d–f. Changing the orientation of the PFUV also changes the velocity contours of the cough vapor and fresh air delivered by the device, as shown in Figure 3g–i. The distribution of the air surrounding Person 2 in Scenario B shows that contours developed on the top of Person 2, compared to scenario A. Therefore, Scenario B reflects better air circulation for Person 2.

**Figure 4:**
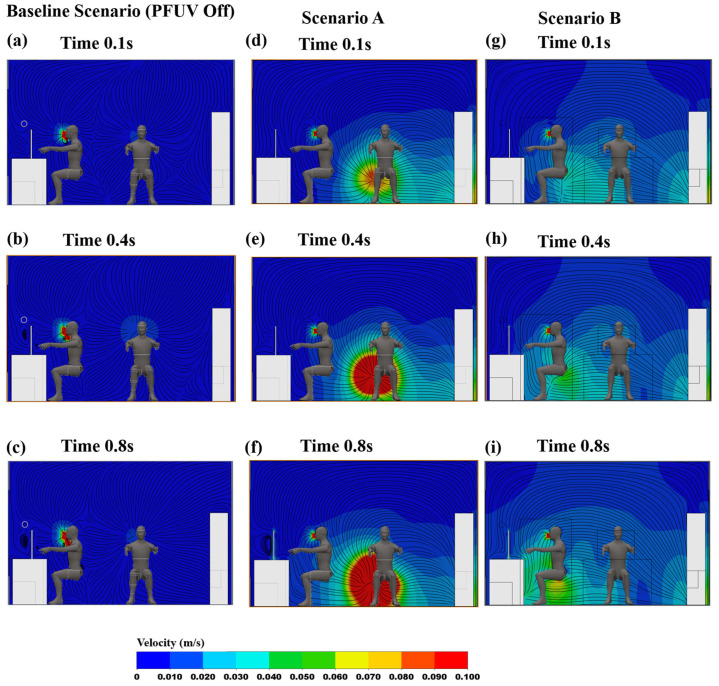
Velocity contours showing Person 1 inhaling: Baseline Scenario (PFUV Off) (**a**) 0.1 s, (**b**) 0.4 s, (**c**) 0.8 s; Scenario A (**d**) 0.1 s, (**e**) 0.4 s, (**f**) 0.8 s; and Scenario B (**g**) 0.1 s, (**h**) 0.4 s, (**i**) 0.8 s.

The results in Figure 3 present a promising improvement, reducing the vapor generated from the cough in the room. We now investigate the contour map for Person 1 (uninfected), again examining along the mid-sagittal plane, to investigate the inhaling (through the nose) of shared air in the room. Figure 4 shows all three scenarios: the device off (Baseline Scenario); the device’s outlet facing Person 2, and away from Person 1 (Scenario A), and the device’s outlet facing Person 1, and away from Person 2 (Scenario B). In Figure 4a–c (the baseline), the vapor cough on that plane is mixing with the inhaled air of Person 1. This can then be compared with the PFUV switched on in both Scenarios A and B. The contours surrounding Person 1 for Scenario A, shown in Figure 4d–f, represent a better distribution of the air in the room, particularly from Person 1′s point of view: note how the air is drawn towards the door outlet.

**Figure 5:**
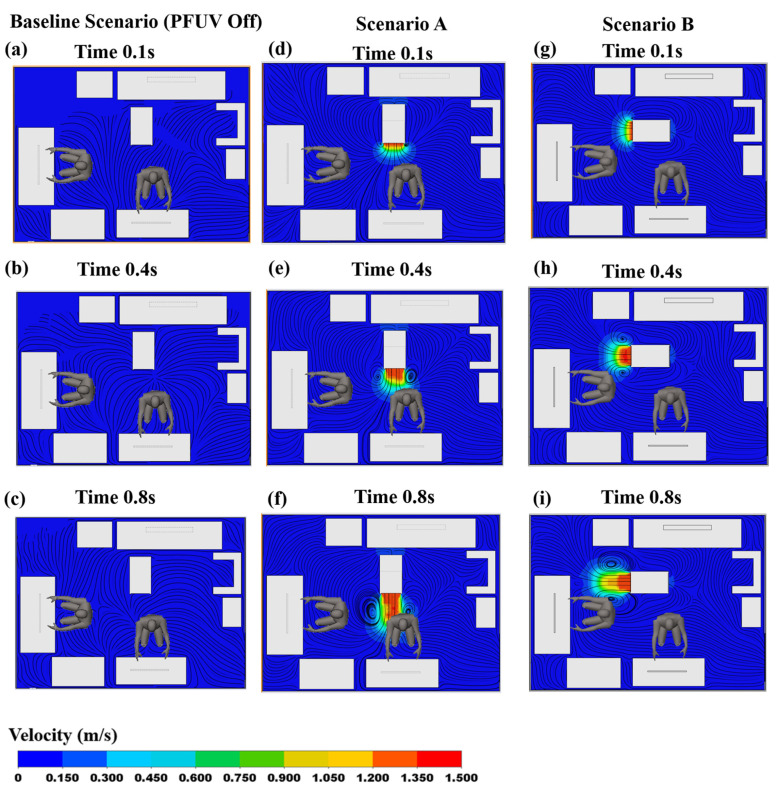
Velocity contours showing the PFUV at 0.4 m height: Baseline Scenario (PFUV Off) (**a**) 0.1 s, (**b**) 0.4 s, (**c**) 0.8 s; Scenario A (**d**) 0.1 s, (**e**) 0.4 s, (**f**) 0.8 s; and Scenario B (**g**) 0.1 s, (**h**) 0.4 s, (**i**) 0.8 s.

To assess the effectiveness of the PFUV, the transverse section plane at 0.4 m of the finished floor height is investigated in terms of velocity contours for the PFUV for all three scenarios (Baseline, A and B), as shown in Figure 5. When the PFUV is switched off, the velocity contours show how the infected occupant distributes the vapor cough in the room, as shown in Figure 5a–c. For the duration of the cough, it is clear by the velocity contour that the stream can reach Person 1. Scenario B, however, shows a circulation of fresh air surrounding Person 1 and a better streamline distribution; therefore, the effectiveness of operating the PFUV in the room is clear, compared to when it is switched off.

**Figure 6:**
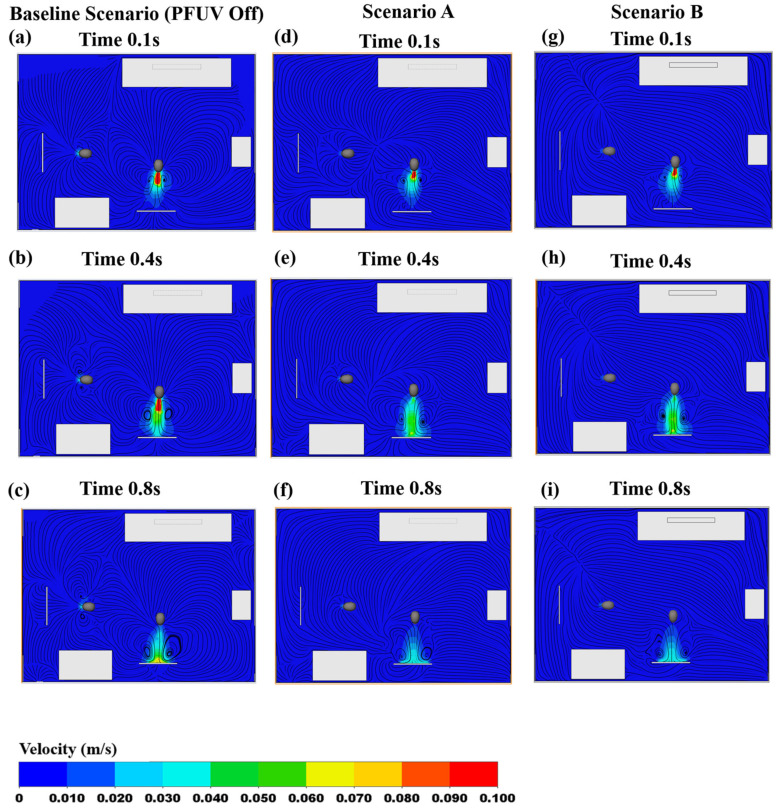
Velocity contours showing Person 1 and 2 at 1.5 m height: Baseline Scenario (PFUV Off) (**a**) 0.1 s, (**b**) 0.4 s, (**c**) 0.8 s; Scenario A (**d**) 0.1 s, (**e**) 0.4 s, (**f**) 0.8 s; and Scenario B (**g**) 0.1 s, (**h**) 0.4 s, (**i**) 0.8 s.

Now, to compare that result to the contour map taken on the transverse plane at a height of 1.2 m, which is the head level of the seated room occupants, when Person 2 is coughing, and Person 1 is inhaling. Figure 6 shows the contour map for the Baseline Scenario compared to Scenarios A and B. Again, Scenario B shows a good outcome for Person 1; however, Scenario A shows good results for Person 2.

### 3.2. Measures of Air Mixing and Thermal Comfort

One of the important measurements for assessing the quality of the ventilation in an indoor environment, such as an office, is the local air quality index (LAQI), when we have multiple fluids mixing, such as the fresh air and the air exhaled by the sick person (vapor). The LAQI reflects how well a system or device removes contaminants from the air at a given point [26]. It is calculated, per Equation (5), as the ratio of the bulk average mass fraction of the contaminant (*C_b_*) in total and the mass fraction of the contaminant at the point being evaluated (*C_m_*). The higher the LAQI, the more effective the system/device is at extracting the contaminant from the air.

(5) $$LAQI=\frac{C_{b}}{C_{m}}$$

**Figure 7:**
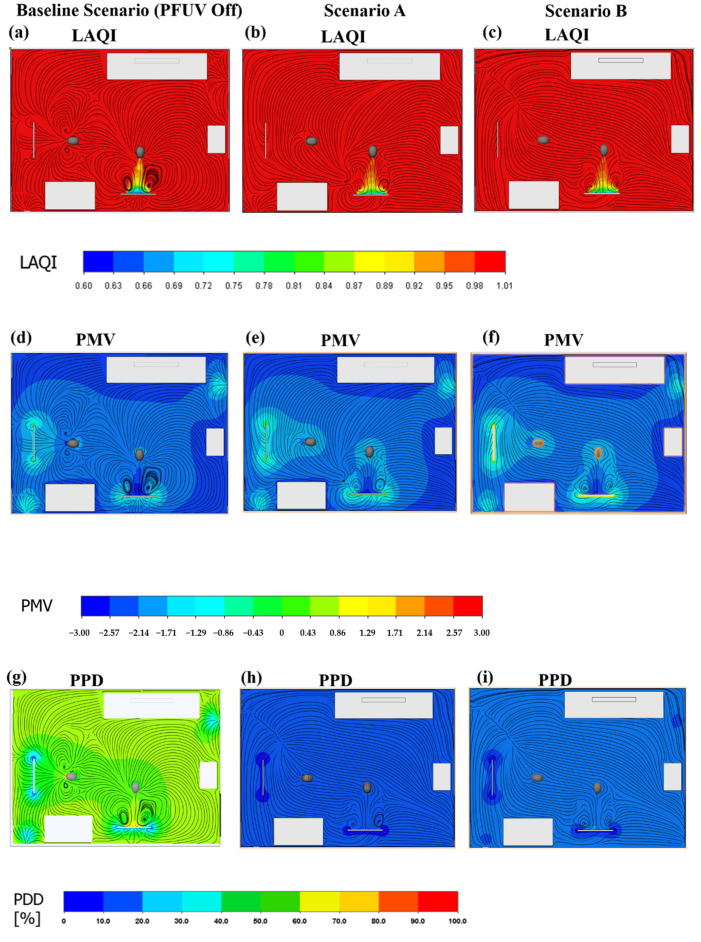
The LAQI for: (**a**) the Baseline Scenario (PFUV Off); (**b**) Scenario A; and (**c**) Scenario B. The PMV for: (**d**) the Baseline Scenario (PFUV Off); (**e**) Scenario A; and (**f**) Scenario B. The PPD for: (**g**) the Baseline Scenario (PFUV Off); (**h**) Scenario A; and (**i**) Scenario B.

Figure 7a–c shows the LAQI contours for the three scenarios under investigation. In both Scenarios A and B, the circulation improves compared to the Baseline Scenario, with the greatest improvement under Scenario B.

A key index for assessing thermal comfort is the predicted mean vote (PMV), which ranges along a scale from −3 (too cold) to +3 (too hot), with zero representing the situation where the person loses no heat to the external environment; this index therefore takes into account biological thermoregulation via the skin (for example, sweating could enable maintenance of heat balance) and is calculated via Equation (6) [27].

(6) $$PMV=\left(0.303\;e^{\left(-0.036\;M+0.028\right)}\right)*L$$

where M is the metabolic rate, which is set to 44 W/m^2^ (metabolic value equals 0.9 for seated and relaxed activity) [5]. Thermal resistance of clothing is set to 0.154 K.m^2^/W (clothing is normalized to 1.00 for typical business wear), and L is the thermal load for person (accounting for surface skin temperature and heat loss via evaporation due to sedentary activities in the office) [27]. The results of the PMV contours are shown in Figure 7d–f. The results show that scenario A reflects better thermal comfort for both Persons 1 and 2.

A related thermal comfort measure, the predicted percent dissatisfied (PPD) index, is considered in this study. The PPD takes into account the PMV, as shown in Equation (7) [27] below, and reflects an estimate of thermal (dis)comfort for room occupants: that is, what percent of occupants are likely to be dissatisfied with the thermal comfort in the room. Due to differences in individuals’ metabolic rates and other factors, PPD is not expected to reach zero (that is, not all occupants in the room are likely to be simultaneously perfectly satisfied with the room temperature). We consider PPD under the three scenarios, as shown in Figure 7g–i.

(7) $$PPD=100-95e^{\left(-0.03353\;PMV^{4}-0.2179\;PMV^{2}\right)}$$

Both the PMV and PPD indices are assessed based on EN ISO 7730 and ASHRAE 55 standards, as well as the EN ISO 12599 and ASHRAE 111 for assessing the PFUV air flow.

**Figure 8:**
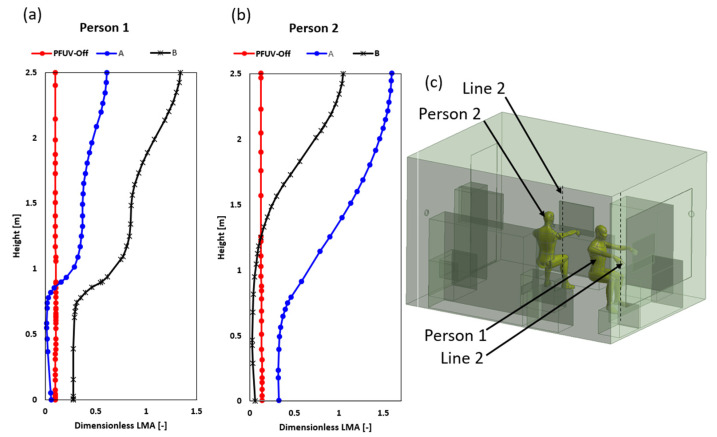
Dimensionless LMA for (**a**) Person 2 (coughing), (**b**) Person 1 (inhaling) and (**c**) the isometric view to the office room.

The last factor assessed in this study is the local mean age (LMA), which reflects the average time τ for the vapor expelled (coughed) to travel from the mouth of Person 2 to the computational volume domain of the office using Equation (8).

(8) $$\overset{3}{\underset{i=1}{\sum }}\frac{\partial }{\partial x_{i}}\left(\rho \tau u_{i}-\left(\frac{\mu }{\sigma }+\frac{\mu_{t}}{\sigma_{t}}\right)\frac{\partial \tau }{\partial x_{i}}\right)=\rho$$

where $x_{i}$ is the *i*-th coordinate, ρ is the density, $u_{i}$ is the *i*-th velocity component, μ is the dynamic viscosity coefficient, $\mu_{t}$ is the turbulent eddy viscosity coefficient, σ and $\sigma_{t}$ are the laminar and turbulent Schmidt numbers. The LMA differs from the LAQI because the former does not measure any specific contaminant; rather, it reflects how frequently the air is circulated. The LMA can then be converted into the Dimensionless LMA, which is the LMA divided by the V/Q ratio, where V is the computational domain fluid volume and Q is the volume flow rate of the air entering this fluid volume from the PFUV device. Therefore, in this study we located two longitudinal poles (shown as the dashed lines in Figure 8 below) in front of each person to determine the dimensionless LMA index under each of the three scenarios.

**Figure 9:**
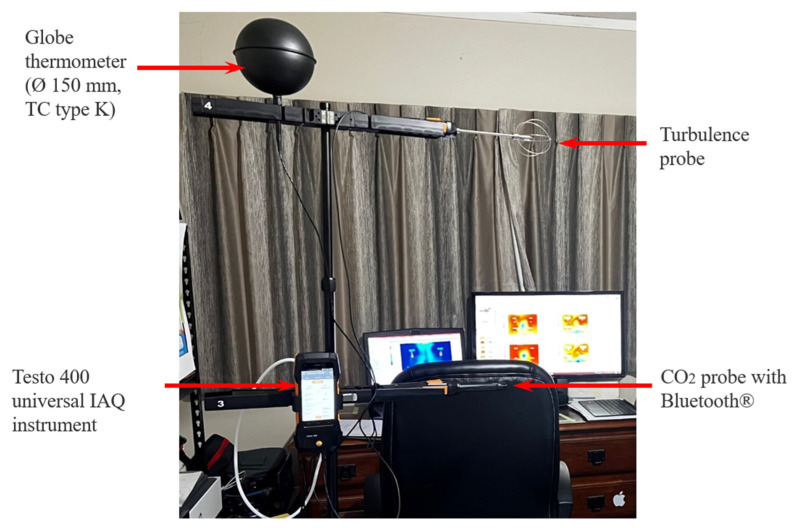
The Comfort Kit with the Testo 400 universal IAQ instrument placed in the office.

The Dimensionless LMA shows how the PFUV improved the local mean age along the height where both Person 1 and Person 2 sit. The results show that the ideal placement of the PFUV is to have the outlet facing the back of whoever is the uninfected person, or Scenario B, to avoid mixing of unfiltered air from one occupant to another. Further to that, the computational study was assessed experimentally using the IAQ and comfort kit with tripod (model: Testo 400), as shown in Figure 9.

**Figure 10:**
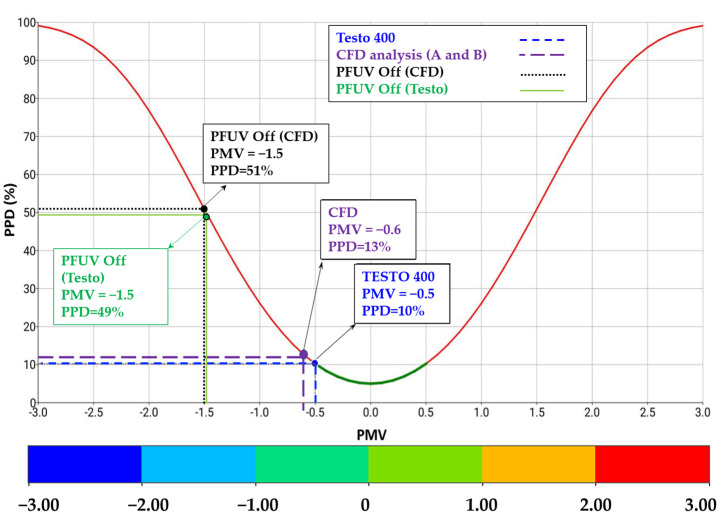
The CFD and experimental validation for PMV and PPD, when the PFUV is off and when the PFUV is on (measured using Testo to validate the CFD results).

Figure 10 shows the CFD results validated against the Testo 400 measurements in terms of PMV and PPD while the PFUV device was operating. The graph shows that, with the PFUV device off (Baseline Scenario) the room was rated to be between cool and slightly cool (at PMV = −1.5) for both the CFD and the Testo 400, whereas the operation of the device (Scenarios A and B) improved thermal comfort to get the room between neutral and slightly cool (up to PMV = −0.5). This means the operation of the device lifts the room temperature to within the accepted range, based on the EN ISO 7730 and ASHRAE 55 standards.

## 4. Conclusions

A common concern over the return to in-person activities in offices, schools and other shared enclosed spaces is the risk posed by infectious occupants sharing the air with those who are uninfected. If a colleague in the office is coughing, other colleagues may feel extremely uncomfortable sharing the office space.

In this paper, both CFD analysis and experimental measurement data are facilitated to determine the mechanism for the dispersion of infectious aerosols and the possibility of diseases spreading in a shared office. The CFD simulation approach is employed to investigate how the behavior of the airflow contours changes when a PFUV dehumidifier device is placed in a room with two occupants, one of which is infectious and coughing. We investigated two different positions for operating the device, and compared the effect on the airflow contours to a baseline situation of no PFUV dehumidifier device operating. Thermal comfort parameters are also investigated computationally and experimentally (using Testo 400 IAQ and comfort kit) and compared to this baseline.

The results show that operating the device has a significant impact on the airflow contours. When the outlet of the PFUV faces the uninfected person, the outcome is better for that person when sharing a space with the infected person, as the air circulation limits exposure to the air coughed out by the infected person. This may serve to allay the concerns of occupants in shared, enclosed spaces: although one occupant did just cough, appropriate placement of the filtration device means the other occupant is not directly inhaling the unfiltered vapor. By reducing exposure to directly exhaled air, this can reduce the risk of inhaling the infected droplets and disease transmission. The device also improves thermal comfort (PMV and PPD) to within the accepted range (PMV = −0.5 and PPD = 10% which fall in the scope of “slightly cool”) based on the EN ISO 7730 and ASHRAE 55 standards, compared to the situation of the device being off, and this is not affected by the placement of the device. Future work will test the impact of air filtration, using the Ultraviolet Germicidal Irradiation lights in the PFUV device.

## Figures and Tables

**Table 1:** Boundary conditions for the CFD modelling of the office meeting room.

Boundary Element	Type	Dimensions L × H × W (mm)	Heat Transfer	Substance Concentration
Office	wall	3900 × 2500 × 2900	Adiabatic, No slip wall	n/a
Laptop 1 + 2	wall	650 × 500 × 20	260 Watt (130 Watt each)	n/a
TV	wall	800 × 600 × 80	130 Watt	n/a
Desk 1	wall	1300 × 800 × 600	Adiabatic	n/a
Desk 2	wall	1200 × 800 × 470	Adiabatic	n/a
Door Outlet	Outlet	800 × 20	Opening Pressure 18 ℃, 50%	Air: 50% Vapor: 50%
Sofa, furniture	wall	-	Adiabatic	n/a
Light	wall	Diameter = 200 mm	40 Watt (LED)	n/a

**Table 2:** Boundary conditions for the CFD modelling of the PFUV, Person 1 and Person 2.

Boundary Element	Type	Dimensions L × H × W (mm)	Heat Transfer	Substance Concentration
PFUV Dehumidifier	wall	640 × 540 × 370	Adiabatic	n/a
PFUV Outlet	Outlet	320 × 160	Velocity = 1.4 m/s 18 ℃, 50%	Air: 100%
PFUV Inlet	Inlet	320 × 140	Velocity = 0.5 m/s	Air: 50% Vapor: 50%
Person 1 (Nose)	Inlet	Diameter = 30 mm	Volume flow rate 0.0015 m^3^/s	Air: 50% Vapor: 50%
Person 2 (Mouth)	Outlet	Diameter = 40 mm	Function (0–22.352 m/s) for 10 s coughing 34 ℃, 100%	Vapor: 100%
Person 1	wall	Area = 0.375 m^2^	Heat Flux = 44 W/m^2^ (For uninfected)	n/a
Person 2	wall	Area = 0.375 m^2^	Heat Flux = 44 W/m^2^ (For infected)	n/a

**Table 3:** Grid independency test and inflation boundary layer.

**Element Size (m)**	**Mesh Elements**	**Vane Anemometer** **(m/s)**	**Outlet Velocity (m/s)** **PFUV Dehumidifier**	**% Error**	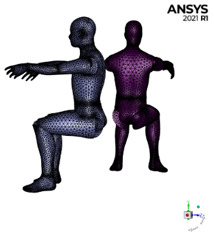
0.03	765,532	1.4	1.475	5.084	
0.02	950,253	1.4	1.415	1.06	
0.01	1,465,125	1.4	1.41	0.709	

## Data Availability

Not applicable.
